# Association of MTMR3 rs12537 at miR-181a binding site with rheumatoid arthritis and systemic lupus erythematosus risk in Egyptian patients

**DOI:** 10.1038/s41598-019-48770-5

**Published:** 2019-08-23

**Authors:** Mahmoud A. Senousy, Hebatullah S. Helmy, Nevine Fathy, Olfat G. Shaker, Ghada M. Ayeldeen

**Affiliations:** 1 Biochemistry Department, Faculty of Pharmacy, Cairo University, Cairo, Egypt; 2 Medical Biochemistry and Molecular Biology Department, Faculty of Medicine, Cairo University, Cairo, Egypt

**Keywords:** Genetics, Rheumatology

## Abstract

Single nucleotide polymorphisms (SNPs) in microRNA-target sites influence an individual’s risk and prognosis for autoimmune diseases. Myotubularin-related protein 3 (MTMR3), an autophagy-related gene, is a direct target of miR-181a. We investigated whether MTMR3 SNP rs12537 in the miR-181a-binding site is associated with the susceptibility and progression of rheumatoid arthritis (RA) and systemic lupus erythematosus (SLE). Overall, 94 patients with RA, 80 patients with SLE, and 104 healthy volunteers were recruited. Genotyping and expression analysis of circulating MTMR3 and miR-181a were performed by qPCR. The autophagic marker MAP1LC3B was measured by ELISA. The rs12537 minor homozygote (TT) genotype was a candidate risk factor of both RA and SLE. rs12537TT was associated with lower serum MTMR3 expression and higher LC3B levels than other genotypes in patients with both diseases. Serum miR-181a expression was higher in rs12537TT carriers than in other genotypes among SLE patients. Serum miR-181a and MTMR3 levels were inversely correlated in SLE but not in RA patients. rs12537TT and serum miR-181a were positively associated with disease severity in both diseases. Our results identify a novel role of rs12537 in the susceptibility and progression of RA and SLE, possibly through impacting the interaction between miR-181a and MTMR3 leading to increased autophagy.

## Introduction

Rheumatoid arthritis (RA) is a disabling autoimmune disease that affects a considerable percentage of the worldwide population^1^. RA is accompanied by constant pain, stiffness, progressive joint destruction, and deformity^2–4^. Systemic lupus erythematosus (SLE) is another systemic autoimmune disorder which comprises the production of multiple autoantibodies against nuclear constituents. SLE presents with a broad spectrum of clinical manifestations that result from the deposition of immune complexes in different organs, mainly the kidney, skin, and joints, causing inflammation and tissue damage^5^.

The exact genesis of RA and SLE remains unknown, but it is thought to occur as a consequence of the interaction of genetic, epigenetic and environmental factors, which contribute to the continuous autoimmune process^6^. To date, studies explaining the genesis of RA and SLE in Egypt are very limited. Hence, the identification of susceptibility genes is urgently needed.

MicroRNAs (miRNAs) are small noncoding RNAs that fine tune gene expression at the post-transcriptional level. miRNAs are implicated in almost all aspects of cellular and humoral immunity^7^. Previous reports have suggested that single nucleotide polymorphisms (SNPs) can create, destroy or modify the efficiency of miRNA binding to the 3′UTR of target genes and ultimately affect an individual’s risk and prognosis of several autoimmune diseases^8,9^.

miR-181a is implicated in haematopoiesis, T lymphocyte maturation as well as B and T cell differentiation^10^. miR-181a overexpression enhanced T cell signalling strength and T cell antigen receptor (TCR) sensitivity to antigens, enabling T cells to respond to antagonists^11^. The upregulation of miR-181a was observed in autoimmune diseases, including SLE and RA^11–15^. Recently, a link between miR-181a and autophagy was demonstrated through its direct target gene, myotubularin-related protein 3 (MTMR3)^16,17^.

MTMR3, a phosphatidylinositol 3-phosphate (PI3P) phosphatase, is an autophagy-related gene involved in negative regulation of autophagy initiation^18^. Autophagy is a destructive mechanism by which cells recycle cellular proteins and cytoplasmic components to generate energy. Autophagy participates in almost every step of innate and adaptive immunity, including pathogen recognition, antigen processing and presentation, and immune cell development, survival, proliferation and function^19^. Dysregulation of the autophagic pathway was recently linked to the pathophysiology of a variety of autoimmune diseases, in particular SLE and RA^19–21^. Indeed, genetic variation in the MTMR3 gene was associated with autoimmune IgA nephropathy in lupus nephritis, which is a major disease manifestation in SLE patients^20^.

The SNP rs12537, which maps to the MTMR3 gene in the 22q12 region, is present in the miR-181a-binding site in the 3′UTR of the MTMR3 gene and was found to be associated with gastric cancer risk^16^. Findings of a genome-wide association study (GWAS) in the Chinese Han population connected the MTMR3 rs12537 with the susceptibility to IgA nephropathy^22^. However, the impact of rs12537 on RA and SLE risk and severity has not yet been investigated.

Recently, many genetic reports have generally agreed on the involvement of shared genetics in autoimmune diseases. This point of view gave us the motive to investigate whether MTMR3 SNP rs12537 is associated with the susceptibility to RA and SLE in Egyptian patients. We also explored the genetic influence of this SNP on serum MTMR3 and miR-181a expression levels, along with the correlations of the investigated parameters with the clinicopathological data of the patients. Given the role of MTMR3 in autophagy^18^, and the previous observation that knockdown of myotubularins, MTMR3 and MTMR14 was associated with increased LC3-II levels, a marker of abnormal authophagy^23^, our aim was extended to clarify the effect of MTMR3 rs12537 on serum levels of the specific autophagic marker human microtubule-associated proteins 1A/1B light chain 3B (MAP1LC3B, referred as LC3B).

## Results

### Demographic, clinical and pathological characteristics of RA and SLE patients

The demographic data from patients and healthy controls are shown in Table 1. Regarding the clinicopathological data, 52.5% of RA patients had a mild/high disease activity score for 28 joints (DAS-28), with 79%, 66%, 21%, and 23.5% of patients had arthritis, deformities, subcutaneous rheumatoid nodules, and extra-articular manifestations, respectively (Table 2). Regarding SLE, 87.5% of patients had an SLE disease activity index-2k (SLEDAI-2K) score ≥6, denoting persistent active disease requiring therapy. More severe disease (SLEDAI-2K score >12) was found in 42.5% of patients. 61.2%, 47.5% and 43.7% of SLE patients had positive anti-double stranded DNA (anti-dsDNA), low complement 3 (C3) and complement 4 (C4) levels, respectively. Proteinuria was observed in 52.5% of patients. In addition, 85% of patients had nephritis of grades II–V (Table 2).

**Table 1:** Demographic data of RA, SLE patients and healthy controls.

Parameter	Healthy controls	RA	SLE	*P* value
	(n = 104)	(n = 94)	(n = 80)	
Sex				0.264
Male, n (%)	16 (15%)	12 (13%)	6 (7.5%)	
Female, n (%)	88 (85%)	82 (87%)	74 (92.5%)	
Age (years)	36.4 ± 7.02	39.47 ± 10.14	32.13 ± 9.1^b^	0.0008
Range	(19–47)	(19–60)	(18–51)	

^b^Significant difference from RA.

**Table 2:** Clinicopathological data of RA and SLE patients.

RA		SLE	
Parameter	RA patients	Parameter	SLE patients
	(n = 94)		(n = 80)
Disease duration (y)	6.24 ± 4.49	ESR (mm)	43.5 ± 30.17
MS (min)	27.23 ± 25.98	Proteinuria (g/day)	1.36 ± 1.5
ESR (mm)	39.64 ± 22.25	>0.5 g/day	42 (52.5)
RF, n (%)		<0.5 g/day	38 (47.5)
Positive	68 (72)	SLEDAI-2K score	11.35 ± 5.4
Negative	26 (28)	>12	34 (42.5)
ANA, n (%)		≤12	46 (57.5)
Positive	12 (13)	Positive anti-dsDNA, n (%)	
Negative	82 (87)	Positive	49 (61.2)
DAS-28 (CRP)	2.95 ± 1.68	Negative	31 (38.8)
Remission <2.6	46 (49)	Low C3, n (%)	
Low disease activity <3.2	8 (8.5)	Yes	38 (47.5)
Mild disease activity 3.2–5.1	30 (32)	No	42 (52.5)
High disease activity >5.1	10 (10.5)	Low C4, n (%)	
VAS (mm)	5.44 ± 3.36	Yes	35 (43.7)
Swollen joint count (SJC)	5.43 ± 5.53	No	45 (56.3)
Tender joint count (TJC)	5.15 ± 5.37	Arthritis, n (%)	
Arthritis, n (%)		Yes	48 (60)
Yes	74 (79)	No	32 (40)
No	20 (21)	Mucosal ulcers, n (%)	
Deformities, n (%)		Yes	36 (45)
Yes	62 (66)	No	44 (55)
No	32 (34)	New rash, n (%)	
Fever, n (%)		Yes	40 (50)
Yes	14 (15)	No	40 (50)
No	80 (85)	Neurological manifestations, n (%)	
Rheumatoid nodules, n (%)		Yes	18 (22.5)
Yes	20 (21)	No	62 (77.5)
No	74 (79)	Nephritis, n (%)	
Extra-articular manifestations, n (%)		Absent	12 (15)
Yes	22 (23.5)	Class II-III	34 (42.5)
No	72 (76.5)	Class IV-V	34 (42.5)

Values are expressed as mean ± SD or number (percentage). Anti-dsDNA, anti-double stranded DNA; ANA, anti-nuclear antibody; C3, complement 3, C4, complement 4; CRP, C-reactive protein; DAS-28, disease asctivity score-28; ESR, erythrocyte sedimentation rate; MS, muscle stifness; RA, rheumatoid arthritis, RF, rheumatoid factor; SLE, systemic lupus erythematosus, SLEDAI-2K, SLE disease activity index-2K; VAS, visual analog scale.

### Association of rs12537 (C/T) with the risk of RA

We performed the SNP genotyping and the subjects’ case-control status was coded. Repeated analyses using the same assay yielded concordance rates of 100%. The minor allele frequency (MAF) in the controls was similar to that in Ensembl GRCh37 release 89, 2017 and gnomAD for the SNP rs12537. The rs12537 genotypes distribution in healthy controls followed the Hardy-Weinberg equilibrium (HWE) (*P* = 0.1).

The genotype and allele frequencies for rs12537 in both RA and SLE patients are shown in Table 3. The rs12537 major C and minor T allele frequencies did not record a significant difference between the patients and controls in both diseases (*P* > 0.05). The frequency of the rs12537 CT genotype was significantly lower in RA patients compared to healthy controls (36.2% vs 57.7%, respectively), and this genotype was associated with decreased RA risk in the codominant [CT vs CC, adjusted OR (95% CI) = 0.5 (0.26–0.97), *P* = 0.001] and the overdominant [CT vs CC + TT, adjusted OR (95% CI) = 0.39 (0.22–0.7), *P* = 0.001] models taking age and sex as covariates. However, the minor homozygote TT genotype was a candidate risk factor of RA when testing the recessive model [TT vs CC + CT, adjusted OR (95% CI) = 2.84 (1.37–5.9), *P* = 0.004].

**Table 3:** Genotype and allele frequencies of MTMR3 rs12537 C/T in RA, SLE and healthy controls.

Genotype and allele	Controls n = 104 n (%)	RA n = 94 n (%)	Crude OR (95% CI)	*P*	Adjusted OR^a^ (95% CI)	*P* ^a^	SLE n = 80 n (%)	Crude OR (95% CI)	*P*	Adjusted OR^a^ (95% CI)	*P*
**MTMR3 rs12537 C/T**											
Codominant											
CC	30 (28.9)	32 (34)	1.00				26 (32.5)	1.00			
CT	60 (57.7)	34 (36.2)	0.53 (0.28–1.02)	0.06	0.5 (0.26–0.97)	**0**.**001**	30 (37.5)	0.58 (0.29–1.14)	0.12	0.7 (0.34–1.43)	0.067
TT	14 (13.5)	28 (29.8)	1.87 (0.83–4.22)	0.15	1.89 (0.82–4.32)	0.13	24 (30)	1.98 (0.85–4.59)	0.14	1.87 (0.77–4.49)	0.16
Dominant											
CC	30 (28.9)	32 (34)	1.00				26 (32.5)	1.00			
CT + TT	74 (71.2)	62 (66)	0.79 (0.43–1.43)	0.43	0.76 (0.41–1.4)	0.37	54 (67.5)	0.84 (0.45–1.58)	0.59	0.96 (0.49–1.87)	0.9
Recessive											
CC + CT	90 (86.5)	66 (70.2)	1.00				56 (70)	1.00			
TT	14 (13.5)	28 (29.8)	2.73 (1.33–5.58)	**0**.**005**	2.84 (1.37–5.9)	**0**.**004**	24 (30)	2.76 (1.32–4.77)	**0**.**006**	2.29 (1.05–4.99)	**0**.**034**
Overdominant											
CC + TT	44 (86.5)	60 (63.8)	1.00				50 (62.5)	1.00			
CT	60 (57.5)	34 (36.2)	0.42 (0.23–0.74)	**0**.**002**	0.39 (0.22–0.7)	**0**.**001**	30 (37.5)	0.44 (0.24–0.8)	**0**.**006**	0.55 (0.29–1.03)	0.063
Log-additive			1.24 (0.84–1.84)	0.28	1.24 (0.83–1.84)	0.29		1.29 (0.86–1.96)	0.22	1.28 (0.83–1.97)	0.27
Alleles											
C	120 (0.58)	98 (0.52)	1.00		—		82 (0.51)	1.00		—	
T	88 (0.42)	90 (0.48)	1.25 (0.84–1.86)	0.31		—	78 (0.49)	1.3 (0.86–1.96)	0.24	—	—

Values are expressed as number (percentage). *P*^a^ adjusted for age and sex in a logistic regression model. *P* values in bold are statistically significant (*P* < 0.05).

### Association of rs12537 (C/T) with the risk of SLE

The rs12537 genotype distribution in patients with SLE showed a pattern similar to that in those with RA. In the overdominant model, CT heterozygosity was a protective risk factor against SLE [CT vs CC + TT, crude OR (95% CI) = 0.44 (0.24–0.8), *P* = 0.006]. However, there was no statistical significance when adjusting for age and sex (*P* = 0.063). Testing the recessive model revealed that the minor homozygote TT genotype was a candidate risk factor for SLE [TT vs CC + CT, adjusted OR (95% CI) = 2.29 (1.05–4.99), *P* = 0.034] taking age and sex as covariates.

### Association of rs12537 with serum MTMR3 and miR-181a expression levels in RA patients

**Figure 1:**
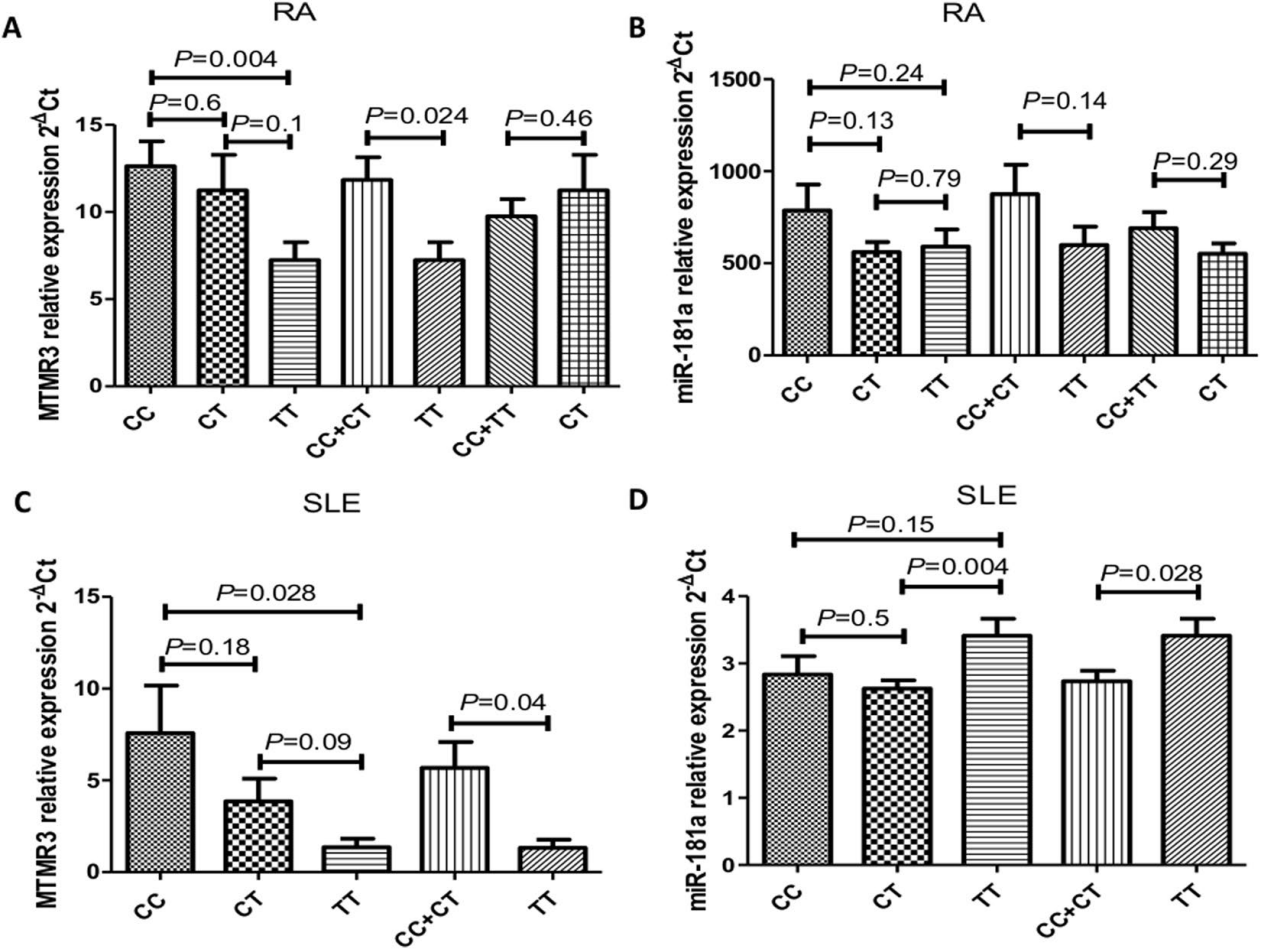
Serum MTMR3 and miR-181a expression levels in RA and SLE patients with different rs12537 genotypes. RA (**A**,**B**) CC = 32, CT = 34, TT = 28, SLE (**C**,**D**) CC = 26, CT = 30, TT = 24. Data are expressed as mean ± SEM.

To explore the impact of rs12537 on MTMR3 mRNA and miR-181a expression, we assessed serum MTMR3 mRNA and miR-181a expression levels in RA patients with different rs12537 genotypes. We found a significantly lower expression levels of serum MTMR3 in patients with the TT genotype than those with the CC (*P* = 0.004) or CC + CT (*P* = 0.024) genotype. TT genotype carriers also demonstrated slightly lower MTMR3 expression levels than CT carriers with no statistical difference (*P* = 0.1). In addition, we failed to find a significant difference in MTMR3 expression when comparing CT vs CC (*P* = 0.6) and CT vs CC + TT (*P* = 0.46) (Fig. 1A). Regarding miR-181a, we failed to record a significant difference in serum miR-181a expression levels among patients with different genotypes in the RA group (*P* > 0.05) (Fig. 1B).

### Association of rs12537 with serum MTMR3 and miR-181a expression levels in SLE patients

We further examined the influence of rs12537 on MTMR3 mRNA and miR-181a expression in SLE patients. We found a significantly lower expression levels of serum MTMR3 in the TT genotype carriers than in those having the CC (*P* = 0.028) or CC + CT (*P* = 0.04) genotype. Subjects carrying the TT genotype also demonstrated lower MTMR3 expression than CT genotype carriers with no statistical significance (*P* = 0.09). MTMR3 expression levels did not differ also in the CT vs CC comparison (*P* = 0.18) (Fig. 1C). Notably, serum miR-181a expression levels were overexpressed in TT genotype carriers compared to those in carriers of the CT (*P* = 0.004), CC + CT (*P* = 0.028) and CC genotypes; however, the difference for the CC genotype was not significant (*P* = 0.15). Serum miR-181a expression levels did not record a significant change between CT and CC genotype carriers (*P* = 0.5) (Fig. 1D).

### Correlation of serum miR-181a with MTMR3 levels in RA and SLE patients

Serum miR-181a and MTMR3 expression levels were negatively correlated in SLE patients (Pearson *r* = −0.3, *P* = 0.036); yet, such correlation was not observed in RA patients.

### Correlation of rs12537 genotypes, serum miR-181a and MTMR3 levels with clinicopathological data in RA patients

In the present study, the prognostic role that might be played by rs12537 SNP, serum MTMR3 and miR-181a expression in RA was examined (Table 4). A positive association of rs12537 with the presence of rheumatoid nodules was observed in the recessive model [TT vs CC + CT, adjusted OR (95% CI) = 3.38 (1.17–9.79), *P* = 0.024]. There was a positive correlation between serum miR-181a and the presence of rheumatoid factor (RF) (*r* = 0.23, *P* = 0.028). However, no correlations were found between serum MTMR3 mRNA expression and clinicopathological data among RA patients.

**Table 4:** Correlation of MTMR3 rs12537, serum miR-181a and MTMR3 expression levels with clinicopathological data of RA patients

Parameter	MTMR3 rs12537 recessive model			MTMR3 rs12537 overdominant model			Serum miR-181a		Serum MTMR3	
	TT (n = 28)	CC + CT (n = 66)	*P* ^a^	CT (n = 34)	CC + TT (n = 60)	*P* ^a^	r	*P*	r	*P*
Disease activity (DAS-28-CRP), n (%)			0.38			0.13	0.073	0.62	−0.043	0.78
Moderate-high (DAS-28 >3.2)	10 (25)	30 (75)		18 (45)	22 (55)					
Remission-low (DAS-28 <3.2)	18 (33)	36 (67)		16 (29.6)	38 (70.4)					
RF, n (%)			0.37			0.58	**0**.**23**	**0**.**028**	0.09	0.55
Positive	22 (32)	46 (68)		24 (35)	44 (65)					
Negative	6 (23)	20 (77)		10 (38)	16 (62)					
ANA, n (%)			0.99				−0.023	0.88	−0.013	0.93
Positive	4 (33)	8 (67)		4 (33)	8 (67)	0.73				
Negative	24 (29)	58 (71)		30 (36.5)	52 (63.5)					
Arthritis, n (%)			0.22			0.51	−0.073	0.63	0.153	0.23
Yes	20 (27)	54 (73)		28 (38)	46 (62)					
No	8 (40)	12 (60)		6 (30)	14 (70)					
Deformities, n (%)			0.24			0.71	−0.1	0.53	0.12	0.43
Yes	16 (26)	46 (74)		24 (39)	38 (61)					
No	12 (37.5)	20 (62.5)		10 (31)	22 (69)					
Rheumatoid nodules, n (%)			**0**.**024**			0.058	0.038	0.8	0.026	0.86
Yes	10 (50)	10 (50)		4 (20)	16 (80)					
No	18 (24.3)	56 (75.7)		30 (41)	44 (59)					
Extra-articular manifestaions, n (%)			0.17			0.94	−0.031	0.84	−0.064	0.67
Yes	4 (18)	18 (82)		8 (36)	14 (64)					
No	24 (33)	48 (66)		26 (36)	46 (64)					

Values are expressed as number (percentage). *P*^a^ adjusted for age and sex in a logistic regression model. *P* values in bold are statistically significant (*P* < 0.05). Correlation was conducted by Spearman correlation. RF, rheumatoid factor; ANA, anti-nuclear antibody.

### Correlation of rs12537 genotypes, miR-181a and MTMR3 levels with clinicopathological data in SLE patients

We also evaluated the prognostic role of the rs12537 SNP, serum MTMR3and miR-181a expression levels in SLE patients (Table 5). rs12537 was positively associated with disease severity (SLEDAI-2K >12) [TT vs CC + CT, adjusted OR (95% CI) = 3.04 (1.1–8.42), *P* = 0.03] and with the presence of low C4 levels [TT vs CC + CT, adjusted OR (95% CI) = 3 (1.1–8.03), *P* = 0.044] in the recessive model. There were significant positive correlations for serum miR-181a expression with SLEDAI-2K scores (Pearson r = 0.318, *P* = 0.04) and with the presence of low C4 levels (r = 0.24, *P* = 0.023). Interestingly, serum MTMR3 expression was negatively correlated with the presence of reduced C3 (r = −0.22, *P* = 0.044) and C4 (r = −0.25, *P* = 0.022) levels. We also recorded a negative correlation between serum MTMR3 expression and urinary protein levels (r = −0.307, *P* = 0.015).

**Table 5:** Correlation of MTMR3 rs12537, serum miR-181a and MTMR3 expression levels with clinicopathological data of SLE patients.

Parameter	MTMR3 rs12537 recessive model			Serum miR-181a		Serum MTMR3	
	TT (n = 24)	CC + CT (n = 56)	*P* ^a^	r	*p*	r	*p*
SLEDAI-2K score, n (%)			**0**.**03**	**0**.**318**^**#**^	**0**.**04**	−0.17^#^	0.13
>12	14 (41.2)	20 (58.8)					
≤12	10 (21.7)	36 (78.3)					
Proteinuria, n (%)			0.1	0.14	0.278	−**0**.**307**	**0**.**015**
>0.5 g/day	9 (21.5)	33 (78.5)					
<0.5 g/day	15 (39.5)	23 (60.5)					
Anti-DNA, n (%)			0.54	0.012	0.96	0.10	0.35
Positive	16 (32.6)	33 (67.3)					
Negative	8 (25.8)	23 (74.2)					
Low C3, n (%)			0.77	0.05	0.64	−**0**.**22**	**0**.**044**
Yes	12 (31.5)	26 (68.5)					
No	12 (28.5)	30 (72.5)					
Low C4, n (%)			**0**.**044**	**0**.**24**	**0**.**023**	−**0**.**25**	**0**.**022**
Yes	15 (42.8)	20 (57.2)					
No	9 (20)	36 (80)					
Nephritis, n (%)			0.56	0.04	0.69	−0.013	0.91
Yes	20 (29.5)	48 (70.5)					
No	4 (33.3)	8 (66.7)					

Values are expressed as number (percentage). *P*^a^ adjusted for age and sex in a logistic regression model. *P* values in bold are statistically significant (*P* < 0.05). Correlation was conducted by Spearman correlation. ^#^Pearson correlation.

### Effect of rs12537 on serum MAP1LC3B (LC3B) in RA and SLE patients

**Figure 2:**
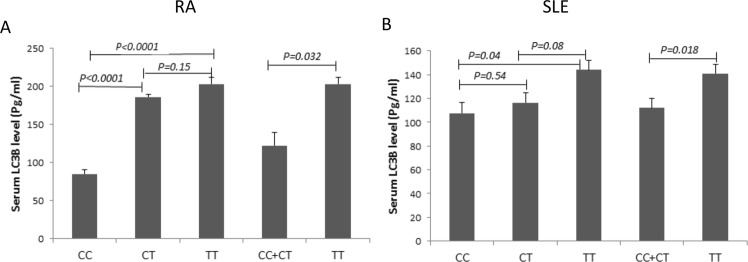
Serum MAP1LC3B (LC3B) levels in RA and SLE patients with different rs12537 genotypes. (**A**) RA, CC = 32, CT = 34, TT = 28, (**B**) SLE, CC = 26, CT = 30, TT = 24. Data are expressed as mean ± SEM.

In both diseases, we found significantly higher levels of the autophagosomal marker LC3B in sera of patients carrying the TT genotype compared to those carrying the CC or CC + CT genotypes (*P* < 0.05). Serum LC3B was also significantly higher in the CT genotype carriers than the CC genotype carriers among RA patients (*P* < 0.0001), but not among SLE patients (*P* = 0.54). There were no significant differences in the CT vs TT comparison in both diseases (*P* > 0.05) (Fig. 2).

## Discussion

**Figure 3:**
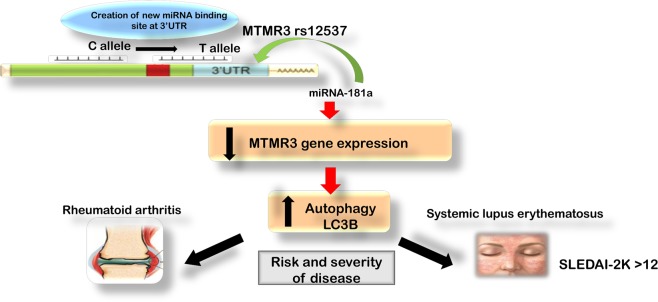
Association of MTMR3 rs12537 at miR-181a -binding site with rheumatoid arthritis and systemic lupus erythematosus risk. MTMR3, myotubularin-related protein 3.

RA and SLE are systemic autoimmune diseases; thus, they may share the same disease mechanisms and susceptibility loci. To our knowledge, we are the first to demonstrate the effects of the MTMR3 rs12537 SNP within the miR-181a-binding site on RA and SLE susceptibility and prognosis. We found that the presence of 2 T risk alleles of rs12537 in the miR-181a -binding site in the 3′UTR of MTMR3 was a candidate for increased risk and severity of both RA and SLE among Egyptian patients recruited in this study, indicating a novel role of this SNP in the genesis and progression of both diseases. These results may partially elucidate the unexplained heritability of these autoimmune diseases. Furthermore, we found that rs12537 TT homozygosity was associated with the lowest expression levels of MTMR3 mRNA among RA and SLE patients which superimposed the highest levels of the autophagy marker, LC3B, linking MTMR3 to the deregulated autophagy in RA and SLE. Notably, the rs12537 SNP was associated with decreased MTMR3 levels and increased gastric cancer risk due to creation of a new miR-181a -binding site^16^. Together, these results confirm the role of this SNP in regulating MTMR3 expression. Additionally, our results suggest that rs12537 could increase the risk and progression of RA and SLE by reducing MTMR3 levels leading to increased autophagy level (Fig. 3).

MTMR3 is a member of the myotubularin-related protein family, which dephosphorylate PI3P; the synthesis of PI3P is required for autophagy initiation and regulation of autophagosome size^18,24^. MTMR3 negatively regulates constitutive autophagy through modulating local PI3P levels^23^. Indeed, targeting the myotubularin family with small inhibitor RNA (siRNA) screen revealed that knockdown of MTMR3 and MTMR14 was associated with altered autophagy markers, including LC3-II^23^. MTMR3 was also reported to modulate pattern recognition receptor (PRR)-induced outcomes and cytokine secretion in inflammatory bowel disease (IBD)^25^. MTMR3 decreased PRR-induced PI3P and autophagy levels in monocyte-derived macrophages, thereby increasing PRR-induced caspase-1 activation, autocrine IL-1β secretion, NF-κB signalling, and, ultimately, overall cytokine secretion^25^. Furthermore, macrophages from MTMR3 rs713875 carriers at risk of IBD exhibited increased MTMR3 expression, with subsequent increased cytokine secretion^25^. Moreover, genetic variants at 22q12 were associated with early-onset IBD in a GWAS, and MTMR3 expression was demonstrated to be significantly reduced in colonic biopsies from ulcerative colitis patients^26^. MTMR3 rs12537 was also associated with the susceptibility to IgA nephropathy; the rs12537 T allele was associated with severe proteinuria^22^. Together, these reports strongly implicate MTMR3 in several pathophysiological mechanisms associated with autoimmune and inflammatory disorders. Nevertheless, the exact function of MTMR3 in RA and SLE remains elusive. The MTMR3 rs9983 high-risk allele at 22q12 showed associations with lupus nephritis, with lower MTMR3 transcription levels were recorded in blood samples with the rs9983 high-risk allele and in renal biopsies from lupus nephritis and IgA nephropathy patients^20^. This finding is consistent with our observation that the rs12537 high-risk allele was associated with SLE risk and reduced MTMR3 levels, along with elevated LC3B levels. This could be clarified by the fact that increased autophagic flux was noticed in T lymphocytes from lupus-prone mice as well as SLE patients^27^, this effect was particularly strong in CD4^+^ T cells. In SLE, proliferating, autoreactive naïve CD4^+^ T cells were shown to undergo enhanced autophagy to supply their metabolic needs^28^. In addition, autophagy activation was also required for survival of autoreactive human and murine lupus B cells and for plasmablast differentiation in SLE^29^. However, to date, the precise role of MTMR3 and its polymorphisms in RA remains unclear. Herein, we demonstrated that the presence of one or two rs12537 risk T alleles (CT or TT) was associated with markedly higher LC3B level than the 2C alleles in RA patients. These results coincided with reduced MTMR3 levels in RA patients carrying the rs12537 TT genotype than other genotypes, strongly implicating a pathologic role of an MTMR3/deregulated autophagy axis in RA. Our results are in agreement with the recent findings that higher levels of autophagosome along with elevated LC3-II levels were observed in peripheral blood mononuclear cells from RA patients compared to healthy controls, and the increased autophagosome was correlated to inflammatory parameters and disease activity in RA patients^30^. Together, these results confirm an intricate relatioship between autophagy and RA.

MTMR3 was picked out as a direct target of miR-181a^16^. Herein, a negative correlation was observed between miR-181a and MTMR3 expression levels in SLE patients. Our results conformed with the results of a previous study, which demonstrated by functional site-directed mutagenesis and luciferase assays that miR-181a suppressed MTMR3 expression by targeting the 3′UTR of the MTMR3 mRNA^16^. Intriguingly, we found that miR-181a was overexpressed in SLE patients carrying the TT genotype compared to those carrying the CT or CC + CT genotype, which paralleled the low MTMR3 expression in this subgroup compared to that in CC or CC + CT genotype carriers. The explanation could be based on that functional assays previously revealed that the replacement of the C allele by the T allele of rs12537 created an additional binding site (at 1653–1673) for miR-181a in the 3′UTR of MTMR3, with perfect complementarity, inducing increased mRNA degradation^16^. Nevertheless, we failed to record either a significant difference in miR-181a expression levels between RA patients with different rs12537 genotypes or a correlation between serum miR-181a and MTMR3 expression in the RA group, suggesting that a more complex mechanism regulates MTMR3 in RA. Indeed, only overexpression of miR-181a significantly reduced MTMR3, whereas knockdown of endogenous miR-181a did not elevate MTMR3 mRNA and protein levels in gastric cancer cell lines, arguing that MTMR3 may be deregulated by other factors in addition to miR-181a^16,17^.

The present study further explored the role of rs12537, serum MTMR3 and miR-181a in SLE and RA prognosis. rs12537 TT genotype and higher levels of miR-181a were recorded in SLE patients with high SLEDAI-2K scores or low complement levels. We also reported a prevalence of rs12537 TT genotype and high serum miR-181a levels in RA patients with rheumatoid nodules and positive RF, respectively. Interestingly, lower serum MTMR3 expression was observed in SLE patients with severe proteinuria or low complement levels. These findings highlight a novel role of rs12537 in the severity and progression of RA and SLE, possibly by reducing MTMR3 through increased miR-181a binding.

Notably, the present data are in accordance with recent studies that demonstrated an association between miR-181a and disease progression in SLE patients^12,15^. Serum miR-181a was highly upregulated in severe SLE cases and showed correlations with anti-dsDNA as well as reduced complement levels (12). In fact, miR-181a is crucial in T lymphocyte maturation and function^10^, and acts as an intrinsic antigen during T cell development^10^. Overexpression of miR-181a increased the stimulation threshold of the TCR in mature lymphocytes and was linked to increased risk of autoimmune diseases^11^. miR-181a also affects T cell function by targeting phosphatases, such as PTPN22 which negatively regulate T lymphocyte activation^11,13^. Indeed, PTPN22 inhibition was implicated in the development and progression of RA^31^. Speculating that miR-181a could enhance autophagy level in T cells by inhibiting MTMR3 may give an additional explanation for the involvement of this miRNA in autoimmune diseases, but this notion should be further investigated.

Six SNPs within the miR-181a-binding sites of miR-181a target genes (RECQL rs13035, KRAS rs9266, TIMP3 rs1143552, EREG rs1460008, IRF5 rs10954213 and MTMR3 rs12537) were previously identified. RECQL rs13035 failed genotyping by PCR-RFLP in a previous study^16^; thus, we excluded it from this study. KRAS rs9266 was also excluded, as it was reported to have little MFE change between wild-type and variant alleles^16^, implying a limited influence on miR-181a binding. TIMP3 rs1143552 has no frequency data in the dbSNP database. EREG rs1460008 has not been previously reported, and IRF5 rs10954213 has been previously demonstrated to be associated with susceptibility to RA^32,33^ and SLE^34^. Herein, we chose MTMR3 rs12537 and demonstrated that rs12537 can predispose to SLE and RA, probably by impacting the interaction between miR-181a and MTMR3 leading to increased autophagy.

While many studies have demonstrated associations of genetic variants related to miRNA genes or their target genes with the risk of RA or SLE^35–37^, our study has several improvements on previous studies in that it is the first to propose MTMR3 rs12537 in the miR-181a-binding site as a novel genetic marker of both RA and SLE risk and prognosis. Our results also shed more light on the shared genetic factors that predispose to these two rheumatic diseases. This confirms that despite the presence of disease-specific gene sets, immune cell types, and organs that drive the distinct phenotypes of both diseases^38^, some features may have a common genetic background. Our study also introduces MTMR3 as a novel biomarker of SLE progression; intensifying the notion that autophagy is linked to the pathogenesis of autoimmune diseases. Additionally, our findings demonstrated that MTMR3 deregulation could be a new mechanism by which miR-181a can regulate immune responses.

Our study has the following limitations. Being a hospital-based study, selection bias was inevitable. Our study was limited to Egyptian patients; this might limit the generalizability of our results; however, homogeneity of the population could be considered a point of strength because it reduces genetic variability. The sample size is relatively small; thus, our results should be cautiously interpreted. Additional large-scale population studies are required to reproduce our findings. Future studies should also take into account the SNP-environment interactions. Nevertheless, we believe that our findings are potentially sound for clinical personalized medicine and have potential implications in genetic counselling, diagnosis and prognosis of RA and SLE. Our findings may also open a new avenue for advanced therapeutic strategies for RA and SLE through targeting MTMR3 and autophagy.

## Conclusions

We are the first to document that the MTMR3 rs12537 TT genotype at the miR-181a-binding site could be employed as a useful genetic marker for higher risk and poor prognosis of RA and SLE. Additionally, miR-181a and its target gene MTMR3 might be involved in SLE progression.

## Subjects and Methods

This case-control study included 94 RA and 80 SLE cases, along with 104 healthy volunteers who were age- and gender-matched. RA and SLE patients were recruited from the rheumatology unit of Kasr Al-Ainy Hospital in Cairo, Egypt, and fulfilled the American College of Rheumatology (ACR 1987) diagnostic criteria. Full patient histories were gathered, and clinical examinations were performed on the patients. The patients were newly diagnosed and treatment-naive. Exclusion criteria included patients who were below 18 years or who had other autoimmune diseases or malignancy, or who were receiving treatment.

Disease activity in RA patients was assessed by DAS-28/C-reactive protein (CRP) as previosly described^39^. Clinical evaluation of SLE disease activity was conducted using the SLEDAI-2K scoring system with 24 descriptor points during 2 visits^40^. SLEDAI-2K scores ≥6 were used to define persistent active disease, while SLEDAI-2K scores >12 were used to define severe disease. Proteinuria was defined as urinary protein levels >0.5 g/24 hr.

We used a questionnaire, to confirm that the healthy volunteers had no history of immunological diseases and were free from other inflammatory or infectious conditions or not being treated for arthralgia, heart failure, renal failure, or autoimmune disease; at the time of recruitment. We obtained a written informed consent from all patients and controls. The study protocol conformed to the ethical guidelines of the Helsinki Declaration and was approved by the ethics committee of the Faculty of Pharmacy, Cairo University.

### Laboratory investigations

Blood samples were collected from all participants. The patients were subjected to routine laboratory investigations, including complete blood count and erythrocyte sedimentation rate (ESR) (mm/1st hr; Westergren method). For RA patients, serum CRP, RF, and anti-nuclear antibody (ANA) levels were performed using commercially available assays. For SLE patients, serum anti-dsDNA, C3 and C4 as well as 24-hr urinary protein levels were determined using commercially available kits. For C3 and C4, the cut-offs provided by the manufacturer were used to define a reduced complement level.

### DNA extraction and genotyping

Genomic DNA was extracted from whole blood samples in EDTA from all subjects using the QIAamp DNA MiniKit (Qiagen, Valencia, CA) according to the manufacturer’s instructions. The yield was measured by a NanoDrop 2000 (Thermo Fisher Scientific, USA). Genotyping was conducted using real-time PCR with the TaqMan allelic discrimination assay using predesigned primer/probe sets for rs12537 (C/T) [C_2615519_1_] (Applied Biosystems, USA) as previously described^41^. Briefly, DNA amplification was performed in a 25 μl volume using a Rotor Gene Q System (Qiagen, Valencia, CA) with the following conditions: 95 °C for 10 min, followed by 45 cycles at 92 °C for 15 s and 60 °C for 90 s.

### Serum miR-181a and MTMR3 assays by RT-qPCR

Total RNA was extracted from serum by a miRNeasy extraction kit (Qiagen, Valencia, CA) and a QIAzol lysis reagent according to the manufacturer’s instructions. The quality of RNA was tested using a NanoDrop 2000 (Thermo Fisher, USA).

For miRNA, reverse transcription (RT) was performed on 100 ng of total RNA in a 20 µl RT reaction using a miScript II RT Kit (Qiagen, Valencia, CA) according to the manufacturer’s instructions. Serum expression levels of the mature miRNA, hsa-miR-181a-5p, were evaluated using a miScript miRNA PCR primer assay and a miScript SYBR green PCR kit (Qiagen, Valencia, CA) according to the manufacturer’s protocols. The housekeeping miRNA SNORD68 was used as the internal control. Real-time PCR was performed in 20 µl reaction mixtures using Rotor Gene Q System (Qiagen) with the following conditions: 95 °C for 30 min, followed by 40 cycles at 94 °C for 15 s, 55 °C for 30 s, and 70 °C for 30 s.

For MTMR3 mRNA, RT was conducted on 100 ng of total RNA in a 20 µl RT reaction using High-Capacity cDNA Reverse Transcriptase kit (Applied Biosystems, USA) according to the manufacturer’s instructions. Serum MTMR3 expression levels were evaluated using GAPDH as an internal control with customized primers and a Maxima SYBR Green PCR kit (Thermo, USA) according to the manufacturer’s protocol. The primer sequences were as follows: MTMR3-forward 5′-AGCAGAGTGGGCTCAGTGTT-3′, MTMR3-reverse 5′-ACTGTCCACGTTTGGT-CCTC-3′, GAPDH-forward 5′-CCCTTCATTGACCTCAACTA-3′ and GAPDH-reverse, 5′-TGGAAGATGGTGATGGGATT-3′. Real-time PCR was performed in 20 µl reaction mixtures using Rotor Gene Q System (Qiagen) with the following conditions: 95 °C for 10 min, followed by 40 cycles at 95 °C for 15 s and 60 °C for 60 s.

The cycle threshold (Ct) is the number of cycles required for the fluorescent signal to cross the threshold in real-time PCR. miR-181a and MTMR3 mRNA gene expression relative to the corresponding internal control was calculated using 2^−∆Ct^, where ∆Ct = Ct gene-Ct internal control.

### Serum LC3 assay

Serum MAP1LC3B (LC3B) was measured by commercially available ELISA kit provided by Cusabio Biotech Co, Ltd, Wuhan, China (Catalog #CSB-EL013403HU).

### Statistical analysis

Statistical analyses for demographic, clinical and gene expression data were carried out using GraphPad Prism-5.0 (GraphPad Software, CA) and the computer program Statistical Package for the Social Science (SPSS, Chicago, IL) software version-15 for Microsoft Windows. Values were expressed as number (percentage), mean ± standard deviation (SD), or mean ± standard error of the mean (SEM) when appropriate. Continuous variables were compared using Student’s t-test or one-way ANOVA followed by Tukey’s multiple comparison test when appropriate. Categorical data were compared by the *X*^*2*^ test. Correlations were determined by Spearman or Pearson correlation when appropriate. For SNP analysis, crude allele and genotype frequencies were calculated and the HWE test was performed for the genotype distribution among controls and cases using the *X*^*2*^ test with one degree of freedom. To test the association of rs12537 with the risk of RA and SLE, unconditional multivariate logistic regression models were used to calculate the odds ratio (OR), 95% confidence interval (CI), and corresponding *P*-value of codominant, dominant, recessive and log-additive models, controlling for age and sex as confounders using SNPStats online software (Inistitut Català d’Oncologia, Barcelona, Spain; https://www.snpstats.net/start.htm) which used the maximum likelihood ratio^42^. In all analyses, the major allele or the common homozygote genotype in the control population was defined as the reference category. Association of the SNP with clinicopathological data was also conducted using logistic regression models controlling for age and sex using SNPStats. In all analyses, *P* < 0.05 was considered significant, with a 95% CI.

## Data Availability

All data generated or analyzed during this study are included in this article.
